# Cell phone addiction and psychological and physiological health in adolescents

**Published:** 2019-02-04

**Authors:** Sehar Shoukat

**Affiliations:** 1 California Institute of Behavioral Neurosciences and Psychology, 4751, Mangels Boulevard, Fairfield, 94534, CA, USA

## ⁯

“These days we have Smartphones, Smart cars, Smartboards, Smart everything, but consider this: if the technology is getting smarter, does that mean humans are getting dumber?” 

Rebecca McNutt

Dear Editor,

Research has been done on smartphone usage and its impact on all adolescents from so many years. It is not a new issue at all. But the increasing trend of cell phone addiction and poor psychological and physiological health of adolescents urged to write this letter. Many studies have been done using different human behavior as dependent and independent variable. Some researcher examined adolescent's physical health or educational performance with smartphone addiction and others analyzed psychological behavior and social relationship with mobile phone addiction. In these articles, some of the latest studies were overviewed. 

The rapid advancement in technology has made many gadgets, a smartphone is one of them (Nishad and Rana, 2016[15]). People spend their time more likely on social media, do business emails, academic search, finding answers to questions, and playing games. Almost 95 percent of Americans own cell phones and 77 percent own smartphones. Around the world, smartphones were used by 1.85 billion people in 2014 which is expected to be 2.32 billion in 2017 and 2.87 billion in 2020 (Cha and Seo, 2018[8]). Such too much dependency makes us “Mobile addictive”. Mobile phones make our lives easier, but on the other hand, it ties us. Mobile addiction not only has physical effects but also psychological and academics effect at the same time. Sleep deficit, anxiety, stress, and depression which are all associated with internet abuse, have been related to mobile phone usage too (De-Sola Gutiérrez et al., 2016[9]). All entities which can stimulate a person can be an addiction. Whenever a habit is converted into an obligation, it becomes an addiction (Alavi et al., 2012[1]). Few researchers believe that smartphone usage and gender are not significantly associated (Nishad and Rana, 2016[15]).

The major question is how do we get to know we are addicted to our cell phone? When a person uses his/her cell phone most of the time, unable to cut back on cell phone usage, using cell phones as a solution to boredom, feeling anxiety or depression when your phone is out of your range, losing your relationships. Research says “when cell phone use becomes an addiction, the behavior becomes stressful”. Salvatore Insiga, a neurosurgeon at Northwell Health's Neuroscience Institute in Manhasset, New York, considered that nonetheless that there is no solid proof between cell phone radiation and tumor risk but the possibility still exists. Adolescents are at high risk of being smartphone addicts (Cha and Seo, 2018[8]). 

Excessive use of smartphone paired with negative attitude and feeling of anxiety and dependency on gadgets may increase the risk of anxiety and depression (Rosen et al., 2013[18], Thomée et al., 2011[20]). Jones (2014[10]) conducted a survey about Elon Students' behavior along with an online survey and found that students seemed to be addicted to their mobile phones. Nevertheless, it was concluded that the excessive smartphone use had a negative psychological effect.

Another research was conducted on mobile phone usage in adolescents. They recruited 439 students, aged 12-17 from Central Switzerland as their sample and distributed a questionnaire among their parents first, then to the children (the procedure was repeated a year later on the same sample). It was concluded that mobile phone usage during night hours was common among youngsters and reported that poor perceived health was shown due to staying up all night. No recordable association was found between memory performance and mobile phones (Schoeni et al., 2015[19]). Reinecke et al. (2017[17]) investigated psychological health effects and stimulator of digital stress. He surveyed 1,557 German internet users aged 14 to 85 and reported that communication load was positively related to perceived stress and had an indirect impact on depression and anxiety too. 

Boumosleh & Jaalouk (2017[6]) investigated whether anxiety and depression independently contributed to smartphone addiction. Their sample was 668 random Lebanese undergraduate students. Their cross-sectional study proposed that depression and anxiety were also a positive predictor of smartphone addiction. They also revealed that with depression scores were a more powerful predictor as compared to anxiety. A study of Brian (2013[7]) subjected “Two days without phone” and revealed that Kenny didn't want to lose his cell ever but Franchesca was happy to not have her cell phone and she decided to give up her phone. Researchers found an intensive increase of cell phone usage among teenagers and the symptoms of depression, suicide risk factors and suicide rate in the year 2012. Cell phone addiction is negatively correlated with academic performance (Ng et al., 2017[14]; Baert et al., 2018[4]; Lepp et al., 2015[11]; Boumosleh and Jaalouk, 2018[5]). Arefin et al. (2017[2]) did a case study on business students in Bangladesh and found that increased impatience and daily life disturbance negatively affected the academic performance of students.

Negi and Godiyal (2016[13]) observed HNBUG-SRT college students while walking around the campus, along with a questionnaire and found 64 % of students used mobile phones in the campus. A randomized sample of 100 students was collected. The survey showed that there were negative psychological effects of smartphone usage on the young generation. They felt depressed and anxious while using cell phones. On the other hand, some youngsters showed relax behavior even without having a cell phone. A study investigated the addiction to the internet and personality traits and found that loyalty, emotional stability, and extroversion were the major predictors of internet addiction (Zamani et al., 2011[21]). Thomée et al. (2011[20]) purposed that high frequency of cell phone use had a risk of mental health outcomes when they had a 1-year followed-up for young students aged 20-24. They concluded that high cell phone usage was associated with sleep deprivation and symptoms of depression for both men and women. 

An online study on Malaysian population stated that heavy mobile phone usage may lead to physiological and psychological complications when a study was conducted on 409 respondents (Parasuraman et al., 2017[16]). A descriptive research suggested that internet addiction is similar to drug addiction except behavioral addiction (internet addiction) doesn't involve a substance. In addition, the physical symptoms are absent in behavioral addiction, but if internet addiction continues, it will undergo the same results as alcohol addiction (Alavi et al., 2012[1]).

Another observational study reports that insomnia may lead to depression. Li et al. (2016[12]) did a prospective cohort and proposed that insomnia and risk of depression are associated. De-Sola Gutiérrez et al. (2016[9]) revealed that the problematic cell phone usage had been associated with sleep deficit, depression, anxiety, and stress. 

Cha and Seo (2018[8]) aimed to examine the predictive factors of smartphone addiction in middle school students in South Korea. Two groups were chosen, one risk group and another normal group. The two groups expressed significantly different results. The predictive factors for smart phone addiction were social networking and awareness of game overuse. A researcher revealed that teenagers who spend more hours on their gadgets are highly likely more at risk of suicide. Another study by Augner and Hacker (2012[3]) examined an association between over usage or dysfunctional usage of cell phones and psychological health. They indicated that low emotional stability, chronic stress, and depression have a correlation with phone usage.

According to latest studies, it is come to know that there are two schools of thoughts. Some researchers believe that there is a positive association between cell phone addiction and the mental health of adolescence and some believe that there is a negative or indirect relation in them. 

It is confirmed that adolescent's mental health and physical health is associated with cell phone addiction. But we cannot say it with 100 % accuracy that mobile phone is the only cause of poor mental or physiological health issues in adolescents. Reviewed articles of this study showed dual results. The result comes in two different schools of thoughts. One opinion emphasizes that cell phone addiction and psychological health has direct relation. Cell phone usage badly affects mental health of adolescents and they look anxious, depressed and angry or sometimes commit suicide. The suicidal rate is increasing in this era. Some studies also showed a positive relation of cell phone addiction and physiological health. 

The other school of thought reveals an indirect relation between cell phone usage and psychological health. They say adolescents use cell phones at night, which leads to insomnia. And insomnia ultimately results in depression, anxiety, and depression. Cell phone addiction has no direct relation to mental health. After reviewing these results, it is concluded that there is a relationship between cell phone addiction and adolescent's mental or physical health whether they have direct or indirect relation. We cannot neglect the relation and its adverse effects on adolescents. It is suggested that more studies should be done in this regard to clarify their nature of relations.
